# Growth factors in the treatment of Achilles tendon injury

**DOI:** 10.3389/fbioe.2023.1250533

**Published:** 2023-09-14

**Authors:** Meina Lin, Wei Li, Xiang Ni, Yu Sui, Huan Li, Xinren Chen, Yongping Lu, Miao Jiang, Chenchao Wang

**Affiliations:** ^1^ Liaoning Research Institute of Family Planning, China Medical University, Shenyang, China; ^2^ Medical School, Shandong Modern University, Jinan, China; ^3^ Department of Plastic Surgery, The First Hospital of China Medical University, Shenyang, China

**Keywords:** Achilles tendon injury, growth factors, tendon healing, clinical trial, combined application

## Abstract

Achilles tendon (AT) injury is one of the most common tendon injuries, especially in athletes, the elderly, and working-age people. In AT injury, the biomechanical properties of the tendon are severely affected, leading to abnormal function. In recent years, many efforts have been underway to develop effective treatments for AT injuries to enable patients to return to sports faster. For instance, several new techniques for tissue-engineered biological augmentation for tendon healing, growth factors (GFs), gene therapy, and mesenchymal stem cells were introduced. Increasing evidence has suggested that GFs can reduce inflammation, promote extracellular matrix production, and accelerate AT repair. In this review, we highlighted some recent investigations regarding the role of GFs, such as transforming GF-β(TGF-β), bone morphogenetic proteins (BMP), fibroblast GF (FGF), vascular endothelial GF (VEGF), platelet-derived GF (PDGF), and insulin-like GF (IGF), in tendon healing. In addition, we summarized the clinical trials and animal experiments on the efficacy of GFs in AT repair. We also highlighted the advantages and disadvantages of the different isoforms of TGF-β and BMPs, including GFs combined with stem cells, scaffolds, or other GFs. The strategies discussed in this review are currently in the early stages of development. It is noteworthy that although these emerging technologies may potentially develop into substantial clinical treatment options for AT injury, definitive conclusions on the use of these techniques for routine management of tendon ailments could not be drawn due to the lack of data.

## Graphical Abstract

**Figure:**
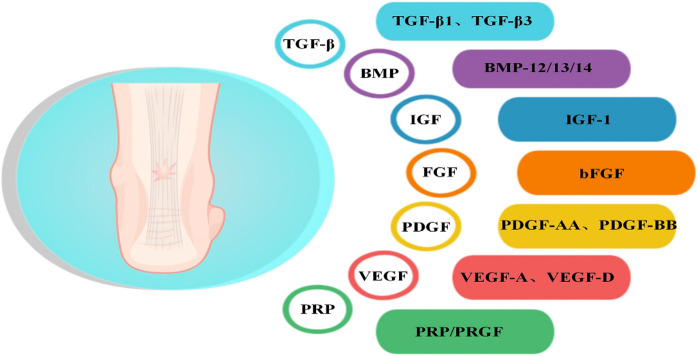

## 1 Introduction

Achilles tendon (AT) injury is a clinically intractable tendon disorder that severely affects patients’ daily activities, imposing a significant clinical burden on healthcare systems worldwide. Naturally healed AT typically have poor quality, as they are prone to developing fibrotic scars and tendon sheath adhesion. These tendons lack the necessary biomechanical properties for proper function and are susceptible to re-injury and re-rupture during exercise and daily activities. This is primarily due to the low number of cells, poor blood supply, and low tendon metabolism (Jiang et al., 2014; Veronesi et al., 2015; Li et al., 2021b). At present, there is a lack of effective clinical treatments to promote functional and anatomical recovery for AT injury (Steinmann et al., 2020). The goal of any therapeutic intervention is to expedite the restoration of complete mechanical strength and deliver a regenerated tendon that closely resembles the original, uninjured state. The healing process of tendons is facilitated and directed by a diverse range of GFs that are produced within the local area. So, recently, the focus has been on the biological pathways by which tendons heal and the GFs involved. GFs involved in cell differentiation, angiogenesis, and extracellular matrix (ECM) production play a vital role in regulating cellular life activities. Therefore, GFs may be promising therapeutics for skin wounds, burns, and nonhealing chronic and diabetic wounds (Legrand and Martino, 2022). GFs also play a critical role in the natural regeneration of tendons as they participate in cell recruitment and stimulation of ECM synthesis. Studies have shown that cells within the paratendinous tissue promote healing in AT injury when stimulated by GFs (Muller et al., 2019). Based on this finding, many studies have focused more closely on how to effectively incorporate GFs into damaged tissues and determine their effects. GFs such as TGF-β, BMP, FGF, VEGF, IGF, and PDGF have shown promising applications in AT repair.

GFs can be administered percutaneously, during open surgery, or through carriers such as scaffolds or coated suture materials. These methods are intended to maintain the concentration of GFs in local areas. However, apart from platelet-rich plasma (PRP), none of these GFs have been successfully implemented in clinical practice due to a lack of compelling preclinical evidence. Single GFs have limited effectiveness in promoting tendon healing, both in laboratory settings and animal models. This suggests that a combination of different GFs/stem cells/ scaffolds may be required to significantly enhance the intricate process of tendon healing. There is ample evidence supporting the use of GFs to improve tendon healing. However, several unresolved issues still exist, such as determining which specific GFs to utilize and the optimal timing and method of their delivery.

In this review, we summarized the advantages and disadvantages of the different isoforms of TGF-β and BMP in AT healing, including GFs combined with stem cells, scaffolds, or other GFs. In addition, we summarized the clinical trials and animal experiments on the efficacy of GFs in AT repair. The findings of studies using PRP had controversial conclusions. Although these emerging basic studies provided extensive information on the therapeutic effects of GFs in AT repair, there is still a long way before they are implemented in clinical use.

## 2 Anatomical characteristics of the AT

**FIGURE 1:**
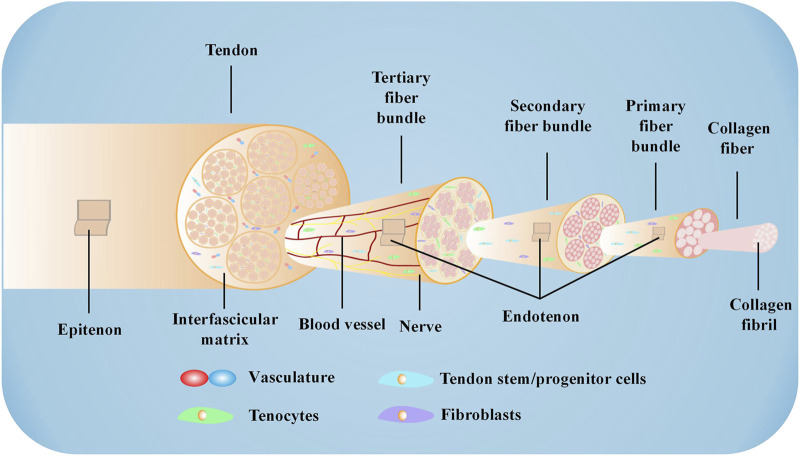
Microstructure of the AT The AT comprises collagen fibers, cells, and a small number of blood vessels. Specific proteases hydrolyze the terminal peptide of procollagen formed by three peptide chains to form tropocollagen. Triple-stranded tropocollagen forms collagen fibril through lateral connections. Collagen fibril further aggregates to form collagen fiber. Collagen fiber converges into a primary fiber bundle surrounded by the endotendon to create a secondary fiber bundle. The secondary fiber bundle also assembles into a tertiary fiber bundle, which is surrounded by the epitenon to constitute the tendon.

The AT is the strongest and largest tendon and is subjected to the highest loads in the human body (McCartney et al., 2019). The tendon has good elasticity and extensibility and plays a vital role in daily life, especially in sports. The AT begins at the musculotendinous junction of the gastrocnemius and soleus muscles and consists of the combined tendons of the soleus, gastrocnemius, and plantaris muscles. The plantaris muscle varies in size and is absent in approximately 6%–8% of individuals (O'Brien, 2005). Generally, the length and thickness of the AT vary between subjects. In adults, the average size of the AT is 15 cm (range, 11–26 cm), and the mean width is 6.8 cm at its origin, 1.8 cm at the midsection, and 3.4 cm at its insertion to the midpoint of the posterior surface of the calcaneus (Doral et al., 2010). Furthermore, the proportion of gastrocnemius and soleus tendons involved in the composition of the AT slightly varies in different individuals (Wasniewska et al., 2022). At approximately 8–10 cm above the termination, the gastrocnemius and soleus tendons fuse completely (Ahmed et al., 1998). Spiral torsion occurs when the tendon fibers move to the termination. The gastrocnemius tendon attaches to the lateral and posterior sides of the calcaneus, while the soleus tendon attaches to the medial and anterior sides of the calcaneus (Dayton, 2017; Winnicki et al., 2020). Studies have confirmed that spiral torsion results in less fiber bending when the ankle is in plantar flexion and less deformation when tension is applied to the AT (Pekala et al., 2017). However, the spiral torsion site, 2–6 cm from the termination, has a relatively poor blood supply and is most prone to rupture and degeneration (Wolff et al., 2012; Nagelli et al., 2022). The AT is encased in the *paratenon*, which is rich in blood vessels, rather than a tendon sheath (Lohrer et al., 2008). A synovial bursa is present between the AT and the skin and calcaneus, which is essential in reducing friction and ensuring relative sliding between tissues (Campanelli et al., 2011). The tendon is also surrounded by the epitenon, a well-defined layer of connective tissue that extends inward to become the endotenon surrounding the fiber bundle (Sharma and Maffulli, 2006). Tendon composition varies slightly in different parts of the body, which may be attributed to the physiological environment (Walia and Huang, 2019). In general, tendons in the human body have similar tissue structures, consisting mainly of collagen and scattered cells (Waggett et al., 1998). The AT mainly contains collagen I (Col I) and collagen III (Col III) (Fukuta et al., 1998; Im and Kim, 2020). Three peptide chains form into soluble tropocollagen after enzymatic excision of the terminal peptide. Tropocollagen forms collagen fibrils through lateral connections. Collagen fibril then aggregates to form collagen fiber. Many collagen fibers gather into bundles, creating primary, secondary, and tertiary fiber bundles (Andarawis-Puri et al., 2015). In addition to collagen fibers, the ECM of the AT includes elastin, fibronectin, decorin, biglycan, and fibromodulin (Walia and Huang, 2019). Tissue-resident tendon cells play a vital role in the composition of AT, exhibiting significant heterogeneity. In mature tendons, tenocytes and tenoblasts make up approximately 90%–95% of the tendon cell population, with tenoblasts being predominant in young tendons. Tissue-resident tendon stem/progenitor cells (TSPCs), which share similar characteristics with MSCs, constitute 1%–4% of the tendon resident cells. Telocytes, found in the equine inter-fascicular tendon matrix near blood vessels, express stem cell markers and are localized near the tendon stem cell niche. Additionally, new populations of tenocytes, including tendon fibroblasts 1 and 2, and junctional fibroblasts, have been recently reported. The remaining cells consist of chondrocytes, synovial cells, capillary endothelial cells, and smooth muscle cells (Sharma and Maffulli, 2005). The heterogeneity of tenocytes, along with the interaction between different cell types in tendon tissue, is crucial for the maintenance and repair of tendon tissue during homeostasis and tendinopathies (Russo et al., 2022). In normal tendons, the collagen fibers are continuous, parallel, and closely arranged (Angrisani et al., 2022). Tenocytes are arranged following the direction of the fibers, extending a few thin-winged pseudopodia embedded in fiber bundles (Millar et al., 2021). The AT exhibits a hierarchical structure (Figure 1) composed of fascicles, fibers, and fibrils. These components are encompassed by a delicate collagen membrane known as endotenon, which houses lymphatics, blood vessels, and nerves. Within this intricate arrangement, certain cells, including tenocytes, fibroblasts, and TSPCs, align themselves along the direction of the fibers.

## 3 Pathophysiology of AT injury

AT injury can lead to loss of tissue integrity and dysfunction, causing pain, swelling, and stiffness (Martin et al., 2018). In tendinopathy, tenocytes become more extended, thinner, smaller in size, and produce less ECM (Millar et al., 2021), and the collagen fibers are broken and arranged irregularly (Maffulli et al., 2008). Simultaneously, inflammatory cells infiltrate the tissue, triggering an inflammatory response (Ye et al., 2023). During tendon injury, the activation of inflammatory mechanisms and the innate immune system is evident within the tendon matrix microenvironment and probably attributed to the dysregulated homeostasis. The resident tenocytes secrete cytokines and chemokines in both autocrine or paracrine manner and can be activated towards an inflammatory phenotype, they influence on the reactions that submerge after tendon damage by communicating with immune-sensing cells, attracting, and activating the infiltrating immune cells into the injury site, or modulating the secreted implicated cytokines (Russo et al., 2022). In addition, supporting tissues including vascular and nervous play an important in modulating the inflammatory response of the injured tissue and in tissue regeneration. So, new nerves are frequently found in diseased tendons (Millar et al., 2021). A normal AT is not rich in blood vessels; however, in AT injury, there are apparent vascular proliferations (Chen et al., 2011). Furthermore, the increased density of Col III in the ECM leads to an abnormal Col I/Col III ratio, thus severely affecting the mechanical properties of the AT (Millar et al., 2017).

## 4 Etiology of AT injury

The AT is the most frequently ruptured tendon in the human body, accounting for 20% of all large tendon ruptures. AT injury is classified into closed and open injuries. Closed injury, such as rupture, tendinitis, and degeneration, is mainly caused by strain and often occurs in the middle, insertion, and peritendinous parts of the AT. Aseptic inflammation accompanied by symptoms such as redness, pain, and weakness may present at the local injured site. Open injury is mainly caused by direct violence, such as chopping and slashing with sharp objects. Other factors that can influence AT injury include age, sex, use of fluoroquinolone antibiotics, and uneven force on the AT during exercise (Seeger et al., 2006; Slane et al., 2015; Ganestam et al., 2016; Tramer et al., 2021). Furthermore, with improved quality of life, obesity has gradually become an essential factor affecting AT injury.

## 5 GFs in AT injury

**FIGURE 2:**
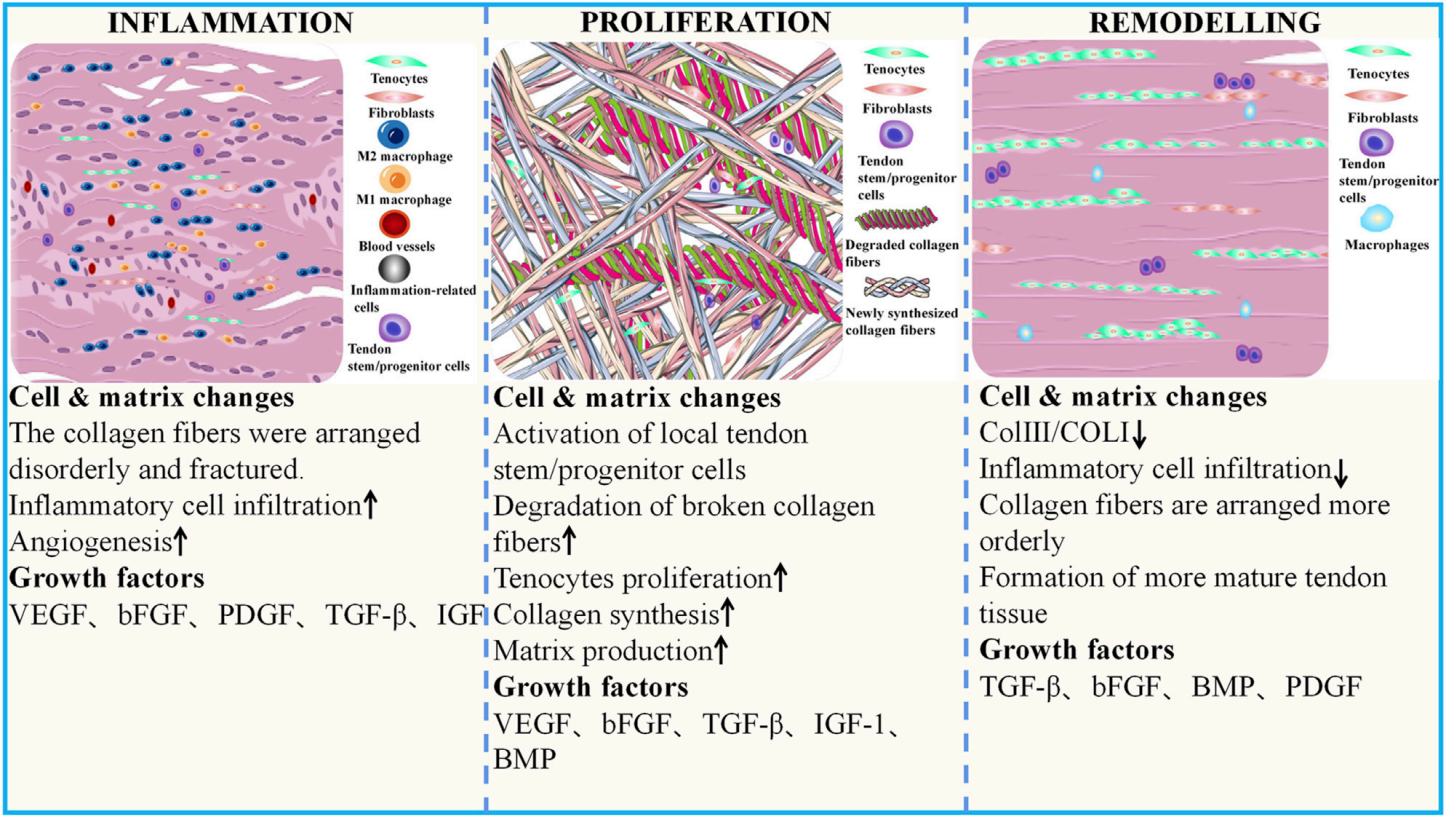
Cell and matrix changes during different phases of AT healing and the GFs involved The recovery process following AT injury is triphasic: acute inflammation, proliferation, and remodeling phases. During the inflammatory phase, collagen fibers are disorganized and broken. There is a considerable appearance of inflammatory cells in the AT and a significant increase in neovascularization. With the activation and proliferation of *in situ* cells, tenocytes and fibroblasts increase significantly, and the recovery phase gradually transitions to the proliferation phase. Here, the content of ECM fractions also gradually increases. The damaged tissue begins to degrade, and new tissues fill the defect site. As rehabilitation progresses, the number of inflammatory cells decreases sharply, the arrangement of collagen fibers becomes more regular, and Col I gradually replace Col III. The biomechanical properties of the AT tissue significantly improve. AT, Achilles tendon; GFs, growth factors.

GFs are a class of cytokines secreted by cells and participate in various biological activities by binding to specific, high-affinity receptors to maintain and regulate the growth and metabolism of the body. A study retrieved 2,332 publications on early tendon development, draw a comparative map of molecules that control tenogenesis, and found several hub genes that plays an important role in tendon development, including TGF-βs, BMPs, FGFs, IGFs (Peserico et al., 2023). These GFs related to tenogenesis paths belonging to macro-categories such as (1) growth, differentiation and survival, (2) morphogenesis and cell motility, (3) nervous system, (4) and endocrine system. GFs such as FGF-4 and TGF-β2, which are critical during embryonic development, influence tendon development. FGF-4 regulates the expression of Scleraxis (Scx), an early marker for tendon cell fate (Glass et al., 2014). As the upstream molecules of Scx, TGF-β and FGF coordinately induce the development of axial and limb tendon progenitors via Scx action. TGF-β plays a central role in tendon development, and TGFB receptors are expressed in tendon progenitor cells, the genetic deletion of TGFBR2 or TGFB2 results in failure of tendon development (Peserico et al., 2023). TGF-β1, -β2, and -β3 have distinct spatiotemporal developmental protein localization patterns in the developing tendon and may probably have independent roles in tendon development (Kuo et al., 2008). TGF-β2 was noted to be tenogenic for tendon progenitor cells at all developmental stages *in vitro* (Titan et al., 2019). BMP-12 guides the expression of Scx, tenomodulin (Tnmd), Col 1, and tenascin-C (TNC) in tendon progenitor cells *in vitro* (Liu et al., 2015b). VEGF signaling is vital during tendon development, specifically within developing tendons under traction, and gliding tendons maintain an avascular zone even from the fetal period (Petersen et al., 2002). GFs such as TGF-β, BMPs, PDGF, VEGFs, FGFs, IGF, and PRP have been studied extensively in various tendon healing models (Table Supplementary S1). These GFs play a role in all three stages of AT healing, which include the inflammatory, proliferative, and remodeling phases (Figure 2). Briefly, In the first phase, the focus is on the inflammatory activities, which involve the infiltration of inflammatory cells and fibroblasts from outside the injury site, and various GFs are released during this phase, including VEGF, PDGF, bFGF, TGF-β, and IGF. The proliferative phase is a crucial stage in the healing process, characterized by the proliferation of fibroblasts, synthesis of ECM, activation and differentiation of TSPCs, and extensive growth of blood vessels and nerves. Various growth factors, including VEGF, BMPs, bFGF, TGF-β, and IGF, play important roles in this phase. In the final remodeling phase, the newly synthesized collagen undergoes rearrangement to form mature tissue. During this phase, GFs such as BMPs, bFGF, TGF-β, and PDGF play a crucial role in guiding and regulating the remodeling process.

### 5.1 TGF-β and AT injury

TGF-β isoforms (TGF-β1, TGF-β2, and TGF-β3) are the prototypical members of the TGF-β superfamily. They are responsible for many cellular activities, including proliferation, differentiation, migration, adhesion, ECM synthesis, immune response, and cell death (Aschner and Downey, 2016; Acharya et al., 2022). The healing response in natural tendon injury is triphasic: inflammation, proliferation, and remodeling phases (Ruiz-Alonso et al., 2021). In the acute inflammation phase, which is the first phase that occurs after tendon injury, proinflammatory factors and inflammatory cell infiltration resist harmful stimuli, mediating the inflammatory response. In contrast, anti-inflammatory and proinflammatory factors act together to control the progression of inflammation (D'Addona et al., 2017). TGF-β expresses at all stages of tendon healing, particularly during the inflammatory and proliferative phases. TGF-β maintains immune homeostasis in several tissues, and the lack of TGF-β exacerbates inflammation, leading to tissue damage and cellular transformation. Its activity decreases with the relief of inflammatory response (Tauriello et al., 2022). Specific genes are selectively expressed during different tendon formation and repair stages to promote normal tendon development (Perucca Orfei et al., 2019). TGF-β isoforms have other effects during wound healing and scarring. While TGF-β3 is a significant inducer of Scx, which is expressed early in tendon development initiating tendon differentiation, it is also an inhibitor of collagen fiber maturation during tenogenic differentiation, especially in the late stage (Perucca Orfei et al., 2019; Zhou et al., 2021a). TGF-β3 alone or combined with other GFs acts as an essential tenogenic inducer in many cell types, such as AD-MSCs (Shojaee et al., 2022), human tenocytes (Tsiapalis et al., 2021), BMSCs (Bottagisio et al., 2017), embryo-derived stem cells (ESCs) (Barsby et al., 2014), tonsil-derived MSCs (TMSCs) (Wee et al., 2022). Contrary to the effects of TGF-β3, TGF-β1 inhibits the expression of Scx, promotes the expression of Tnmd, which is a marker of mature tendon cells, and accelerates tendon development (Hyun et al., 2017).

Proper application of TGF-β1 at the early stage of injury can reduce inflammation and accelerate wound healing (Sun et al., 2021). Collagen content and cross-linking are significant determinants of tendon structural integrity and function. Col Ⅰ and Col Ⅲ are the main contents of the AT. TGF-β1 may participate in matrix remodeling by modulating collagen synthesis (Sun et al., 2015). AT injury treated with TGF-β1-bone marrow mesenchymal stem cells (BMMSCs) healed more rapidly and completely. TGF-β1 accelerates collagen protein synthesis, cross-link formation, and matrix remodeling in tendon healing, thus enhancing mechanical strength (Hou et al., 2009b). Low-magnitude, low-frequency 10 Hz vertical vibration training enhances TGF-β1 expression, subsequently increasing the expression of Tnmd and synthesis of Col Ⅰ and increasing AT stiffness in rats. This treatment improves tendon properties and minimizes the risk of ligament/tendon reinjury during rehabilitation (Chen et al., 2018b). A study demonstrated that in patients with AT rupture, TGF-β1 and VEGF 3 expressions significantly increased 3 months after treatment and significantly decreased 6 months after surgery. TGF-β1 and VEGF expressions decreased after surgery with improvement in the efficacy; therefore, TGF-β1 and VEGF can be considered as observational indexes and predictors of clinical efficacy in patients with AT rupture before and after surgery (Cui et al., 2019). Collagen sponges loaded with GFs, such as basic FGF (bFGF), BMP-12, and TGF-β1, implanted in a rat with transected AT showed a rapid increase in mechanical strength and faster tendon remodeling (Majewski et al., 2018). The TGF-β1 and TGF-β3 changes in the coursing of AT healing in a rat model was higher than that in the sham operation group at all-time points (2w, 4w, 6 weeks after injury), reaching its peak at 2 weeks, decreased at 4 weeks, and significantly reduced at 6 weeks after the operation. Therefore, the expression levels of these two factors may be used as indicators to determine the degree of recovery following an AT injury (Wu et al., 2021). Rat muscle biopsies transduced with recombinant adenovirus-TGF-β1 were transplanted to surgically transected AT of recipient animals, which accelerated the healing, and the repair tissue gained nearly normal histological appearance at 2 weeks post operation (Majewski et al., 2012). In addition, the treatment notably alleviated the inflammatory responses *in vivo* via downregulation of IL-1β, TNF-α, and IL-6 and promoted the tube formation in tissues through upregulating VEGF, bFGF, TGF-β1, and CD31 (Gong et al., 2022).

Although TGF-β fosters recovery after AT injury, its overexpression can result in abnormal deposition of ECM proteins, leading to tissue fibrosis, induced excessive scar hyperplasia in the injured area, altered tissue structure, and decreased anatomical function (Schroer and Merryman, 2015). In addition, elevated active TGF-β promotes ectopic bone formation. Huang et al. demonstrated that injured AT of rats transfected with TGF-β short hairpin RNA using ultrasound-targeted microbubble destruction technique attenuated AT adhesions and scar formation, and the decreased TGF-β helped to alleviate tendon adhesion by reducing the number of inflammatory cells (Hung et al., 2022). Although attenuating the effect of TGF-β1 can diminish the grading of adhesions, the ultimate strength of repaired tendons was significantly impaired (Zhou et al., 2013).

In contrast, TGF-β3 is well known for its antifibrotic effects. Jiang reported that adding TGF-β3 to tenocytes significantly downregulated the expression of Smad3 and upregulated the expression of Smad7 (Jiang et al., 2016b), minimizing extrinsic scarring via antagonizing the TGF-β1/Smad3 signaling pathway (Jiang et al., 2016a; Deng et al., 2017). This result provides a new therapeutic approach for reducing scar tissue and promoting tendon healing. Cetik et al. used PLGA-b-PEG NPs [poly (lactic-co-glycolic acid)-b-poly (ethylene glycol) nanoparticles (NPs)] loaded TGF-β3 as a sustained-release system to treat rat models with unilateral AT transection and demonstrated that TGF-β3 may positively affect AT midsubstance repair, especially during the remodeling phase, and the NP form will achieve better outcomes (Cetik et al., 2022).

TGF-β2 was the only isoform detected in tenocytes within the fibrillar matrix, and its expression was significantly higher in patient tendons compared to normal cadaver tendons. This elevated presence of TGF-β2 in pathological AT suggests its potential role in regulating cellular activity during the advancement of this disease (Fenwick et al., 2001). TGF-β2 and TGF-β3 expressions fluctuated during bone formation. TGF-β2 was significantly upregulated during HO formation in animal model for HO induced by Achilles tenotomy in rats (Lin et al., 2010). The zinc finger transcription factor (EGR1) plays a role in the production of type I collagen in postnatal tendons. In a rat model of AT injury, the application of EGR1-producing MSCs resulted in an increase in the formation of tendon-like tissues. This effect is partially mediated by TGF-β2 (Guerquin et al., 2013). Mohawk (Mkx) is expressed in developing tendons and is an important regulator of tenogenic differentiation, its expression level was dramatically lower in human tendinopathy tissue and it is activated at specific stages of tendon development. Mkx dramatically upregulated Scx through binding to the TGF-β2 promoter. Mkx activates Scx and tendon ECM genes expression partially through direct activation of TGF-β2 (Liu et al., 2015a).

In conclusion, the TGF-β isoforms have different effects during AT healing and scarring (Table 1 **)**, even if the same subtype has a double-sided effect. However, further studies regarding the effective ways to use these factors to promote AT healing and prevent scar formation or their role as indicators of the degree of recovery following injury are warranted.

**TABLE 1:** The advantages and disadvantages of TGF-β isoforms in AT repair.

TGF- β isoforms	Advantages	Disadvantages	Summary
TGF- β1	①Increases the collagen synthesis You et al. (2020).	Its overexpression will result in abnormal deposition of ECM proteins, alteration in tissue structure and anatomical hyperplasia Schroer and Merryman (2015), lead to tissue fibrosis, excessive scarring, and HO Wang et al. (2018b).	TGF-β isoforms function in the regulation of collagen synthesis in tendon fibroblasts. Proper application of TGF-β1 at the early stage of injury can reduce inflammation and accelerate wound healing. Attenuating the effect of TGF-β1 at the late stage will diminish the extent of scar formation. TGF-β3 exerted antagonistic effects to TGF-β1.
	②Promotes tube formation Gong et al. (2022).		
	③Increases Tnmd, Col I and the AT stiffness Chen et al. (2018a).		
	④Reduces the inflammatory response Pakshir and Hinz (2018).		
	⑤Increases the proliferation, migration, and differentiation of TSCs Li et al. (2021a).		
TGF- β2	① Increases Scx, Col I and Eln in TPCs and MSCs Brown et al. (2014); Brown et al. (2015).	Induces HO formation Lin et al. (2010).	
	② Induces Tnmd expression, alters cadherins and connexin-43 protein expression Theodossiou et al. (2019).		
	③ Increases paracrine factors and ECM molecules in tendon healing and promotes tenocytes migration Koch et al. (2022).		
	④ Promotes tenogenesis Chien et al. (2018),Tnmd-expressing mature tenocytes differentiation Yoshimoto et al. (2022) and collagen synthesis Font Tellado et al. (2018)		
TGF- β3	① Induces Scx expression, initiates tendon differentiation Hyun et al. (2017).	NA	
	② Anti-fibrotic effects, minimizes scarring via antagonizing the TGF-β1/smad3 signaling Jiang et al. (2016b); Deng et al. (2017).		

NA, no data available.

### 5.2 BMPs and AT injury

BMPs constitute the largest subdivision of the TGF-β family with nearly 30 different proteins (Lowery and Rosen, 2018). However, significant differences among BMPs regarding their effects on AT repair exist. BMP-1 is significantly upregulated at each time point of traumatic heterotopic ossification (HO). On the contrary, BMP-4 is significantly downregulated in the early stages of traumatic HO. This indicates that BMP-1 plays an essential role in the formation of traumatic HO, whereas BMP-4 plays a protective role in the early stage of HO (Yu et al., 2021a). The BMP-2/4/7 expression in Sprague-Dawley (SD) rats AT increased with aging, especially BMP-2, and this expression remained consistent with the increasing trend of HO and osteogenesis-related gene expression in the tissue. BMP-4/7 might play an essential role in forming ectopic ossification at the early stage and a weaker function at the late phase (Dai et al., 2020). BMP-2 negatively regulates the expression of Prospero homeobox protein 1, thereby inhibiting the formation of lymphatic endothelial cells, aggravating the inflammatory response, and promoting the formation of HO (Dunworth et al., 2014).

**FIGURE 3:**
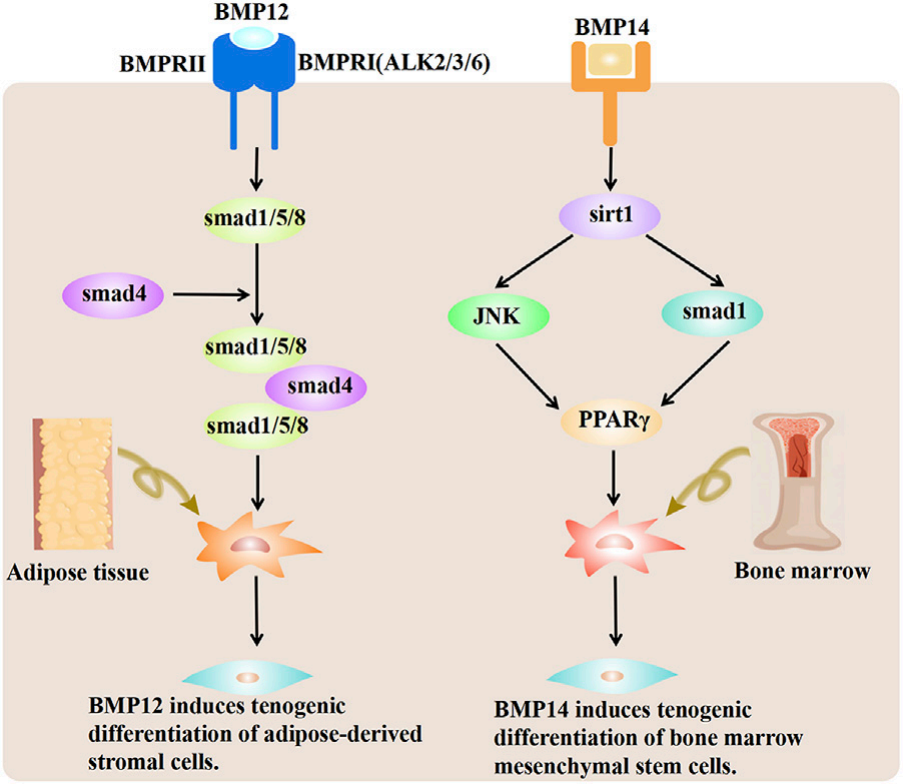
BMP-12 and BMP-14 mechanisms promoting tenogenic differentiation of MSCs. BMP-12 induces tenogenic differentiation of ADMSCs via the Smad1/5/8 pathway. BMP-12 triggers the phosphorylation of Smad1/5/8 in ADMSCs. The effect is likely conveyed by type I receptors ALK2/3/6. BMP-14 induces the deacetylation of PPARγ through activation of the Sirt1-Smad1/JNK pathway. Deacetylated PPARγ further promotes the tendon differentiation of BMMSCs. BMP-12, bone morphogenetic protein 12; BMP-14, bone morphogenetic protein 14; MSCs, mesenchymal stem cells; ADMSCs, adipose tissue-derived mesenchymal/stromal stem cells; BMMSCs, bone marrow mesenchymal stem cells.

BMP-12/13/14 can induce tendon differentiation *in vivo*. BMP-12/14 induces tendon differentiation of adipose tissue-derived mesenchymal stromal/stem cells (ADMSCs), and BMP-12 induces tendon differentiation of ADMSCs via the Smad1/5/8 pathway in a dose- and time-dependent manner (Shen et al., 2013) (Figure 3). BMP-14 may induce the tenogenic differentiation of BMMSCs via the Sirt1-JNK/Smad1-PPARγ signaling pathway (Wang et al., 2018a) (Figure 3). Adenovirus-mediated gene therapy of BMP-14 expedited tendon healing in SD rats model with transected AT; BMP-14 significantly increased the Col II expression and enhanced the mechanical properties of the AT (Wang et al., 2018a). In another study, BMP-12 was tethered on a book-shaped decellularized tendon matrix and implanted into a rat AT defect model, which proved more beneficial than autograft for AT healing (Xiao et al., 2021). Exogenous BMP-7 in a rat AT has been shown to enhance fibro-chondrocyte differentiation of tendon cells, induce ectopic cartilage formation, promote meniscus regeneration, and prevent cartilage degeneration (Ozeki et al., 2013). Biopsies of autologous skeletal muscle transduced with Ad. BMP-12 and surgically implanted around experimentally transected AT in a rat model improved and accelerated tendon healing and influenced early tissue regeneration, leading to quicker recovery and improved biomechanical properties of the AT (Majewski et al., 2008). Lee et al. showed that BMMSCs implanted into an animal model after loading onto a three-dimensional collagen sponge scaffold and induction by BMP-12 promoted tendon-like tissue formation (Lee et al., 2011). In addition, the number of tenocytes increased, which were arranged regularly along the tension axis. Engineered tendon matrix containing BMMSCs and BMP-13 implanted in an AT injury model significantly improved the fiber alignment, increased the tensile modulus, and increased the ultimate load of the AT (Jiang et al., 2016a). Park et al. showed that 100 ng/mL of BMP-14 significantly promoted the proliferation of ADMSCs and expression of the tendon marker genes Scx, Tnmd, and TNC (Park et al., 2010). Although BMPs combined with stem cells can promote AT healing, differences in the therapeutic effects of different sources of stem cells on AT injury exist. BMMSCs, ADMSCs, and synovial membrane-derived MSCs (SMMSCs) induced with BMP-12 all showed similar fibroblast morphology and expressed typical tendon marker genes, such as Scx, TNC, and Tnmd (Dai et al., 2015). Nonetheless, BMMSCs showed superior tendon differentiation ability, followed by SMMSCs, while ADMSCs showed the least tendon differentiation ability. However, compared with BMMSCs, BMP-14-induced muscle-derived MSCs were more effective in promoting tendon healing (Ozasa et al., 2014). The advantages/disadvantages of BMPs are presented in Table 2.

**TABLE 2:** The advantages and disadvantages of BMPs isoforms in AT repair.

	Advantages	Disadvantages	Summary
BMP-1	NA	①Degrades collagen precursor, inactivates BMP antagonists, activates TGF-β1, and induces HO Yu et al. (2021a).	Overall, different BMPs play different roles in various stages of AT healing. BMP-2/12/13/14 are potent inducers of tenogenic differentiation in MSCs, BMP-1/2/7/14 induce HO, otherwise, BMP-4 inhibits HO.
BMP-2	① Improves tendon healing and biomechanical parameters Pelled et al. (2012).	Promotes chondrogenic differentiation TSCs Xu et al. (2016), and induces HO *in vivo*.	
	② Increases human tendon cell growth and viability Arslan et al. (2016).		
	③ Mediates TC differentiation and tendon-like tissue formation of MSCs Ker et al. (2011); Noack et al. (2014).		
	④ Promotes the woven bone in tendon-bone junctions and increases the mean maximal load Kim et al. (2011).		
BMP-4	① Enhances tendon-to-bone attachments healing, promotes the regeneration of fibrocartilaginous enthesis and mineralization Chen et al. (2021).	NA	
	② Inhibits HO Yu et al. (2021a).		
	③ Enhances human TCs growth and viability Arslan et al. (2016).		
BMP-7	① Enhances human TCs growth and viability Arslan et al. (2016).	Induces fibro-chondrocyte differentiation of TCs, and HOOzeki et al. (2013).	
BMP-12	①Induces MSCs to differentiate into tenocytes and promotes tendon healing Xiao et al. (2021).	NA	
	②Improves collagen organization, reduce adhesions, decreases cell numbers Chamberlain et al. (2015).		
	③Regulates ingrowth at the enthesis		
	④Accelerates tendon remodeling by increasing Col1 and shifting fibroblasts to fibrocytes.		
	⑤Improves tendon healing, leading to regenerates Majewski et al. (2018).		
BMP-13	①Induces MSCs to differentiate into tenocytes.	NA	
	②Induces elastin and Col I, resulting in stronger tendons;		
	③Increases tendon fibroblasts proliferation, matrix remodeling, tissue regeneration, COL1, COL3 expression, and the strength in healing tendon Eliasson et al. (2008); Mikic et al. (2009).		
BMP-14	①Induces MSCs to differentiate into tenocytes Bottagisio et al. (2017); Wang et al. (2018a)	Induces cartilage formation Rickert et al. (2001)	
	②Increases tendon resistance, stiffness, tensile strength, and neotenocytes nummber at the site of healing Bolt et al. (2007); Rickert (2008).		

NA, no data available.

In summary, BMP isoforms exhibit different expression patterns in various stages of AT repair and play various roles in the AT healing process. BMP-2, -12, −13, and −14 are potent inducers of tenogenic differentiation in different MSCs, promoting AT recovery. BMP-1, -2, -7, and -14 induce HO, whereas BMP-4 inhibits HO. Therefore, to enhance AT healing and decrease HO, the effective use of BMP isoforms is important.

### 5.3 IGFs and AT injury

The IGF family plays a critical role in normal growth and development. It comprises (i) ligands (IGF-I, IGF-II, and insulin), (ii) six well-characterized high affinity binding proteins (IGF binding protein [IGFBP]-1–6), (iii) IGFBP proteases, and (iv) cell surface receptors that mediate the biological functions of IGFs (IGF-1 receptor [IGF-1R], IGF-2R, and insulin receptor substrates) (Stuard et al., 2020). IGF-1 is critical for tenocyte migration, division, matrix expression, collagen synthesis, phenotypic maintenance, and tendon repair after injury (Muller et al., 2015). A study showed that after regular training, the cross-sectional area of tendons increased significantly in men than in women, which may be related to IGF-1 secretion and sex; the cumulative effect of the amount of IGF-1 secretion makes men more superior to women due to motor-adapted hypertrophy of the AT (Astill et al., 2017). IGF-1 accelerates tendon recovery and promotes tendon-derived stem cell (TDSC) phenotype proliferation and maintenance (Holladay et al., 2016). IGF-1 combined with TGF-β1 induces ADMSCs to differentiate into stable tenocytes (Schneider et al., 2011). IGF-I injections stimulated collagen synthesis in Ehlers-Danlos patients (Nielsen et al., 2014). Tang et al. showed that IGF-1 significantly improved the mechanical properties of the AT, which may be attributed to the promotion of collagen synthesis by IGF-1 (Tang et al., 2015). IGF-1 signaling is required for proper tendon growth in response to mechanical loading through a coordinated induction of collagen synthesis and cell proliferation. Disser et al. demonstrated that IGF-I induces tenocyte proliferation via the RAS/RAF/MEK/ERK signal transduction pathway and stimulates ECM protein synthesis via the PI3K/Akt signaling pathway (Hortensius and Harley, 2013; Disser et al., 2019). After AT injury, resident cells, such as macrophages and mastocytes, recruit immune cells from circulation to build a protective system. Tenocytes participate in the inflammatory response and tissue remodeling through autocrine or paracrine secretion. While low-level inflammation clears damaged tissues and promotes tissue recovery, a too-strong inflammatory response can lead to tissue fibrosis, resulting in poor remodeling and seriously affecting the healing of the AT. IGF-1 may alleviate the functional impairment after AT injury by reducing the inflammatory response (Kurtz et al., 1999). As there are few studies on the effects of IGFs on AT healing, it would be interesting to analyze whether different concentrations of IGF-1 can generate different results.

### 5.4 FGFs and AT injury

FGFs are broad-spectrum mitogens and play a critical role in development, metabolism, and tissue homeostasis by regulating a wide range of cellular functions, including migration, proliferation, differentiation, and survival. FGFs exert pleiotropic effects by binding to and activating high-affinity tyrosine kinase receptors (FGFR) (Xie et al., 2020). Fibrin clots and vitamin C produce a more robust tendon structure and better quality tendon healing in the surgical treatment of AT ruptures, which may be attributed to the proper stimulation of FGFs secretion during the first phase of AT injury (Celik et al., 2021).

FGF2, also known as bFGF, promotes cell proliferation and migration and accelerates wound healing (Zhang et al., 2018a). bFGF stimulates collagen production, tendon development, tenocyte proliferation, and tendon tissue differentiation and promotes the expression of a series of GF genes in AT repair. bFGF stimulates ECM secretion and slows down ECM degradation by upregulating the expression of tissue inhibitors of metalloproteinase (MMP) (Tang et al., 2016). A study demonstrated that human TDSCs transfected with a lentivirus carrying the FGF2 gene promoted the expression of Col 3A1 and Scx *in vitro* (Guo et al., 2020). In addition, the rat AT defect model transplanted with FGF2-hTDSCs demonstrated more ECM production and a more orderly arrangement of collagen fibers. Hyun et al. found that FGF2 dose-dependently increased Scx and Tnmd expressions, which are markers of tendinogenesis, in human periodontal ligament stem cells. Simultaneously, FGF2 counteracted the inhibition of early tendinogenic marker expression by TGF-β1 and attenuated the ossification effect of BMP-2/4 (Hyun et al., 2017). The lasting time of GFs used in tendon injury is key in determining their effects. Tissue-engineered scaffolds can sustainably release GFs to promote cell infiltration and tissue formation. bFGF-loaded multiscale fibrous scaffolds (polycaprolactone [PCL]/Col/bFGF) with appropriate porosity provide physical and biochemical cues to facilitate tenocyte proliferation and differentiation. *In vivo* study of cell-seeded scaffold after dynamic stimulation in the AT defect model showed tendon tissue regeneration, with aligned collagen morphology within 12 weeks of implantation, and inhibition of scar formation, thus playing an essential role in maintaining the tendon’s ultimate tensile strength (UTS) (Jayasree et al., 2019). Chen et al. demonstrated that FGF gene expression was associated with improved patient-reported outcomes and suggested that FGF expression in surgical biopsies could potentially be used as a predictor for healing (Chen et al., 2021).

HO, an extraskeletal bone formation, is a common complication after trauma; however, its underlying mechanisms remain unclear. The degree of ossification depends on the level of damage/inflammation. Zhang et al. revealed that conditional knockout FGFR3 in Col2^+^ cells promote acquired HO development. Knockdown of FGFR3 in lymphatic endothelial cells inhibits local lymphatic formation in a BMPR1a-pSmad1/5-dependent manner, exacerbating inflammatory levels in the repaired tendon, leading to HO. Therefore, activating FGFR3 in lymphatic endothelial cells may be a therapeutic strategy to inhibit HO formation by increasing local lymphangiogenesis (Zhang et al., 2021). However, further studies evaluating its plausibility and the role of FGF in AT healing are warranted.

### 5.5 PDGFs and AT injury

PDGF, a serum-derived GF, is essential for the maturation of multiple cells. It includes four isoforms: PDGFA, PDGFB, PDGFC, and PDGFD. These isoforms dimerize to form homodimers PDGF-AA, PDGF-BB, PDGF-CC, and PDGF-DD and heterodimers PDGF-AB (Paolini et al., 2022). PDGF receptor (PDGFR) is a transmembrane glycoprotein belonging to class III receptor tyrosine kinases, including two subtypes, PDGFRα and PDGFRβ, which can polymerize with each other to form three dimers: PDGFRαα, PDGFRαβ, and PDGFRββ (Guerit et al., 2021). Five distinct PDGFs can bind to one or both PDGFRs in a dimeric state contributing to the normal development of different tissues. PDGF-AA, which only activates PDGFRα, drives both tenogenesis and fibrosis. Tubulin polymerization-promoting protein family member 3-expressing (Tppp3+) cell population can generate new tenocytes and self-renew upon injury. PDGF-AA induces new tenocyte production while inactivating PDGFRα in Tppp3+ cells block tendon regeneration. However, PDGF-AA can also act on fibro-adipogenic progenitors and lead to tendon fibrotic scar formation (Harvey et al., 2019). Therefore, studies regarding the use of PDGF-AA to accelerate AT recovery while reducing scar formation are worth conducting.

PDGF-BB is considered the universal isoform of PDGF and approved by the Food and Drug Administration because of its ability to bind to all three PDGF receptors and trigger different signaling pathways (Evrova and Buschmann, 2017). PDGF-BB expresses predominantly during tendon healing. It induces tissue repair through its generic chemotactic, mitogenic, and angiogenic properties and synergistic actions with other GFs. The supplementation of tenocyte culture with PDGF-BB increased tenocyte proliferation in a rabbit AT model, leading to the upregulation of fibronectin, biglycan, and TNC in the cultured tenocytes (Evrova et al., 2020). Chen et al. demonstrated that recombinant human PDGF-BB promoted hADMSC proliferation via the miR-363/PI3K/Akt pathway. PDGF-BB and hADMSC can improve the biomechanical indices of Achilles tendinitis separately, such as stiffness, stress, and maximum load-to-failure, and upregulate Col Ⅰ, Scx, and TNC expressions. PDGF-BB and hADMSC can also enhance these effects further (Chen et al., 2018b). The combined application of two or more GFs can overcome the defects of a single GF and even have a cumulative effect, thereby promoting healing. PDGF-BB combined with growth differentiation factor-6 (GDF-6) can stimulate the tenogenic differentiation of ADMSCs, with results better than that of a single GF (Younesi Soltani et al., 2022). ADMSCs cultured with GDF5/PDGF before implantation can promote tendon repair by improving cellular proliferation, differentiation, tenogenesis, vascular infiltration, proteinogenesis gene SOX9 expression, and tissue remodeling (Fitzgerald et al., 2021).

Degradable biological scaffolds are often used to deliver GFs continuously at the site of injury for long-term effects. Evrova et al. found that PDGF-BB loaded with polyester urethane scaffold significantly increased the expression of α-smooth muscle actin, promoted the proliferation of tenocytes, and improved the UTS of the tendons, with no significant local scar hyperplasia (Evrova et al., 2020). Kang et al. suggested that the long-term local PDGF delivery by porous microspheres modified with heparin has a great potential to enhance tendon healing in a rat model of Achilles tendinitis by suppressing inflammation responses (Kang et al., 2019). PDGF-AA-modified poly (lactide-co-glycolide) acid (PLGA) electrospun fibers (PLGA-PDGF-AA) effectively promoted tendon healing by stimulating collagen synthesis, deposition, and mechanical strength of tendon tissue (Wang et al., 2022). The sustained delivery of PDGF-BB via an electrospun DegraPol tube to the wound site in a full-transection rabbit AT model accelerated tendon wound healing by causing a more uniform cell distribution, with higher proteoglycan content and less fibrotic tissue (Meier Burgisser et al., 2020). Liu et al. constructed a multilayer composite membrane and a GF sustained-release system conforming to the tendon-healing cycle by coating two surfaces of freeze-dried amnions with PCL nanofibers (Liu et al., 2020). In the study, the rabbit tendon injury model treated with the composite membrane effectively isolated the exogenous adhesion tissue and promoted endogenous tendon healing by slowly releasing TGF-β1, bFGF, VEGF, and PDGF and regulating the ERK1/2 and SMAD2/3 pathways.

### 5.6 VEGFs and AT injury

The VEGF family includes VEGF-A, VEGF-B, VEGF-C, VEGF-D, VEGF-E, placental GF, and endocrine gland-derived VEGF. VEGF promotes endothelial cell mitosis, improves vascular permeability, and stimulates cell migration. In addition, VEGFs binding to VEGF receptors (VEGFR) promote tyrosine kinase enzyme activation and several intracellular signaling pathways (Melincovici et al., 2018).

Although tendons are relatively hypovascular, they become hypervascular during injury and degeneration, and this may be attributed to the formation of new blood vessels in the injured tissue. Neovascularization facilitates healing by controlling the immune response, delivering oxygen and nutrients, removing waste products, and transporting regulatory factors. Vascular ingrowth is necessary for tendon healing; prolonged hypervascularization following tendon injury may not be beneficial. VEGF plays a vital role in angiogenesis. Cui et al. found that during the inflammatory phase of tendon healing, M2 macrophages can release VEGF and promote endothelial cell sprouting, contributing to angiogenesis (Cui et al., 2022). As a potent regulator of angiogenesis, VEGF dose-dependently promotes tenocyte proliferation, enhances the expression of tendon-related genes (Kraus et al., 2018), facilitates the formation of microvessels, and improves the UTS of the AT when used appropriately. A study has shown that reducing VEGF- signaling leads to tendon healing (Tempfer et al., 2018). VEGFR1, VEGFR2, and VEGFR3 expressed in murine and human tendon cells *in vivo* and VEGFR1, VEGFR3, and VEGF-D expressed in tenocytes respond to inflammatory stimuli and injury both *in vitro* and *in vivo* and can affect tenocyte proliferation, stromal disruption, cell migration, and degenerative changes in the AT (Tempfer et al., 2022). After a tendon injury, tenocytes secrete hypoxia-inducible factor 1 (HIF-1) in response to mechanical overload and hypoxia. Simultaneously, HIF-1 promotes VEGF expression, resulting in increased vascularization and accelerated tendon healing (Tempfer and Traweger, 2015). A study showed that the serum TGF-β1 and VEGF expressions in patients with AT rupture significantly increased 3 months and significantly decreased 6 months after surgery, as compared to results before treatment. Therefore, TGF-β1 and VEGF may be considered observational indexes and predictors for clinical efficacy in patients with AT rupture (Cui et al., 2019). A study indicated that compared with normal controls, VEGF and corresponding protein levels significantly downregulated in the healing AT of type 2 diabetic rats 2 weeks post injury; the vascular remodeling ability decreased, and the recovery was slow (Ahmed et al., 2014). Therefore, upregulation of VEGF levels may be a strategy to improve tendon repair in diabetic rats during the early stages of AT injury.

VEGF-111 is a biologically active and proteolysis-resistant splice variant of VEGF-A. Local injection of VEGF-111 significantly improved the UTS of the healing ATs 15 and 30 days after surgery and the mechanical stress in the late phase (30 days) of the repair compared to the control group (Kraus et al., 2014). Many studies linked heparin injection to poor outcomes in rat AT repair due to its role in the inhibition of thrombin activity and anticoagulation effect. However, suture loading with heparin has achieved good results. Poly-l-lactic acid/polyamide sutures loaded with heparin can reduce inflammation and accelerate the healing and regeneration of the AT by promoting VEGF secretion (Ye et al., 2018).

Vascularization in healthy tendons is low; however, the production of new vessels after an injury is not necessarily a sign of functional tissue repair. Instead, it may be associated with degeneration. Some studies have demonstrated that antiangiogenic treatment in tendon models may cause improvements in tissue organization and mechanical properties. Bevacizumab, an antiangiogenic drug that is a recombinant humanized monoclonal antibody blocking VEGF-A signaling, can alter tendon vascularity and dose-dependently improve tendon healing (Riggin et al., 2019). In addition, the drug can significantly improve tendon healing in a rat model by reducing angiogenesis, cross-sectional area, stiffness, and Young’s modulus, thereby improving matrix organization and increasing maximum load and stress. The gait pattern of the rat model also improved (Tempfer et al., 2018). ADMSC transplantation into injury sites during tendon repair in a mice model significantly increased VEGF and CD31 positive vessels, repairing the tendinopathy and preventing HO (Kokubu et al., 2020). VEGF can also cause scarring by stimulating the formation of a hypofunctional vascular connective tissue at the injury site, disrupting the typical molecular structure. Using VEGF to promote recovery from AT injury can be complicated. Hence, further studies are needed to assess whether VEGF is appropriate for tendon healing.

### 5.7 PRPs and AT injury

PRP is an autologous blood product containing high concentrations of GFs and cytokines, such as PDGF, IGF, VEGF, and TGF. It alters the biological processes in many pathogeneses, promotes injured tissue regeneration, accelerates anabolism, and improves healing by stimulating cell proliferation, migration, and angiogenesis (Boesen et al., 2017; Bennell et al., 2021; Park et al., 2021; Xu et al., 2021). Depending on their leukocyte and fibrin content, PRPs can be classified into pure PRP (P-PRP), leukocyte-rich PRP (LR-PRP), pure platelet-rich fibrin (P-PRF), and leucocyte- and platelet-rich fibrin (L-PRF) (Dohan Ehrenfest et al., 2014). Recent clinical and experimental studies have demonstrated the successful application of PRP in treating chronic tendinopathy and acute tendon injury. PRP can promote tissue recovery in the early phase of tendon healing by stimulating tendon cell proliferation and collagen production while inhibiting apoptosis and macrophage infiltration (Yu et al., 2021b). Zou et al. injected PRP into the paratenon sheath and around the ruptured tissue of patients with AT tendon rupture after surgery, and the results demonstrated that PRP improved the short- and mid-term functional outcomes after surgical repair (Zou et al., 2016). Platelets are a major high mobility group box1 (HMGB1) source. Platelets HMGB1 within PRP play a vital role in tendon healing by decreasing inflammation, increasing local HMGB1 levels, and recruiting stem cells to the wound area, suggesting that the efficacy of PRP treatment for tendon injuries in clinics may depend on platelets (Zhang et al., 2021).

Compared to LR-PRP, leukocyte-poor PRP (LP-PRP) is a better choice for treating tendinopathies. The high amounts of proinflammatory factors and catabolic enzymes in leukocytes exacerbate the inflammatory response after injury. LP-PRP may promote AT healing by modulating the balance of MMPs and tissue inhibitors of metalloproteinases and reducing proinflammatory catabolic cytokines release. In addition, the leukocytes present in LR-PRP may provoke further chronic inflammation (Yan et al., 2017). PRP combined with a variety of therapies can enhance AT healing. MSCs combined with PRP significantly increased the inflammatory cell density, mean maximum breaking force, and tendon strength force (Uyar et al., 2022). The primary use of glucocorticoid enhanced the regenerative effects of PRP in early inflammatory tendinopathy (Ruan et al., 2021). However, PRP combined with eccentric training in chronic Achilles tendinopathy proved more effective in improving activity levels and reducing pain, tendon thickness, and intratendinous vascularity (Boesen et al., 2017). A study indicated that PRP injection may be an intensive treatment for patients with AT rupture (Padilla et al., 2021). However, studies have shown that the application of PRP in the nonsurgical treatment of AT rupture showed no special clinical effect or functional improvement and is not as effective as percutaneous fixation in reducing pain (Boesen et al., 2017; Kearney et al., 2021; Kirschner et al., 2021). This difference in results may be attributed to varying treatment regimens. Nonetheless, despite the controversial results of PRP use for tendinopathy, it remains the most used biological treatment (Kirschner et al., 2021).

In conclusion, PRP is the only GF used in clinical studies because of its apparent advantages, such as self-sufficiency, convenient extraction, and high safety. However, the results are inconsistent, and studies regarding using PRP for routine management of tendon ailments are limited to allow definitive conclusions.

### 5.8 Combination of GFs with other GFs/stem cells/ scaffold in AT injury

As mentioned above, various GFs activate cellular processes, ECM deposition, and tissue regeneration during the different phases of tendon healing. Specific GFs critical in tendon healing are TGF-β; BMP-12, -13, −14; bFGF; PDGF; IGF-1; and VEGF; however, some GFs present complex effects on AT recovery. To improve AT healing and reduce complications, most studies mainly use GFs, often combined with scaffolds or stem cells, to surgically create tendon defects (Table 3). Several types of stem cells, including BMSCs, AD-MSCs, TDSCs, ESCs, and terminated differentiated cells (Muscle cells), were utilized in combination with GFs and scaffolds for AT treatment. These stem cells were derived from various sources. The clinical application of ESCs has been limited due to ethical concerns. On the other hand, MSCs are extensively utilized in musculoskeletal repair due to their ability to self-renew and differentiate into various mesoderm-derived tissues, such as bone, cartilage, muscle, tendon, and fat. BMSCs and AD-MSCs are the two main types of MSCs used in AT injury, and both have shown significant improvements in AT healing in animal models. However, AD-MSCs have certain advantages such as ease of harvesting, ready availability, and low donor site morbidity. On the other hand, the use of BMSCs is limited due to the painful technique of bone marrow aspiration, which can also cause donor site morbidity (Pillai et al., 2017). TDSCs, or tendon-derived stem cells, possess a similar proliferative capacity to other types of stem cells. However, TDSCs have demonstrated higher clonogenicity, faster proliferation, and increased expression of TnmD, Scx, and Col1A1 compared to BMSCs (Tan et al., 2012). TDSCs may be a more favorable cell source for musculoskeletal tissue regeneration. However, obtaining autologous TDSCs without causing donor site morbidity can be challenging. Many results indicated that combining GFs with SCs or scaffolds may be a promising approach to treating AT injury. However, further studies investigating whether the combination of GFs/SCs/scaffolds can achieve the best efficacy with the least adverse effects in AT injury are warranted.

**TABLE 3:** GFs combined with other GFs/SCs/Scaffolds in the treatment of AT injury.

	GFs	Combined application	Outcomes	Animal models/defect type	References
GFs/other GFs	TGF-β	TGF-β+ IGF-1	Reduces Col Ⅲ, increase Col Ⅰ, ML, and ensile stress.	Rat/Transect	Xiang et al. (2018)
	BMP	TGF-β1+BMP-12+bFGF	Enhances AT healing.	Rat/4-mm defect	Muller et al. (2019)
		+paratenon			
	FGF	bFGF+VEGF165+rPDGF	Promotes blood vessel densities**.**	Rabbit/Transverse hole (3 mm diameter)	Hou et al. (2009b)
GFs/SCs					
	TGF-β	TGF-β1+BMSCs	Promotes Col Ⅰ synthesis, ECM remodeling and fiber bundles.	Rabbit/Transverse hole (3 mm diameter)	Hou et al. (2009b)
		TGF-β1+Muscle cell	Accelerates AT healing, restore mechanical strength, and increases tendon thickness.	Rat/Transect	Majewski et al. (2012)
		TGF-β1/VEGF (165)	Improves AT healing and biomechanical properties, leading to quicker recovery.	Rabbits/Transverse hole (3-mm diameter)	Hou et al. (2009b)
		+BMSCs			
	BMP	BMP-2/Smad8+MSC	Increases the stiffness, elastic modulus, and functional recovery.	Murine/2-mm full thickness defect	Hoffmann et al. (2006), Pelled et al. (2012)
			Induces tendon regeneration.	Rat/3-mm-long partial-resection defect	
		BMP-12+skeletal muscle	Improves AT healing and biomechanical properties, influences early tissue regeneration, leading to quicker recovery.	Rat/Transect	Majewski et al. (2008)
	FGF	bFGF+hTDSCs	Increases ECM, orderly collagen fibers, Col Ⅲ, Col Ⅰ and biomechanical properties.	Rat/3 mm gap	Guo et al. (2020)
		bFGF+MSC	Increases Col Ⅰ production.	Rat/punch (2.4 mm)	Kraus et al. (2014); Kraus et al. (2016)
	PDGF	PDGF+GDF5+ADSCs	Improves cellular proliferation, tenogenesis, and vascular infiltration, tendon repair.	Rat/cut transversely 1.5 cm	Fitzgerald et al. (2021)
	VEGF	TGF-beta1/VEGF (165) +BMSCs	Accelerates tendon healing, improve biomechanical properties of AT.	Rabbits/A complete transverse hole (3 mm diameter)	Hou et al. (2009a)
		VEGF (165)+TGFβ1	Promotes angiogenesis of the reconstructed ligament and its mechanical properties.	Rabbits/ACL reconstruction	Wei et al. (2011).
		+BMSCs			
	PRP	PRP	Increases tensile strengths, Col I, FGF, and VEGF, decrease the	Rabbits/Incision	Uysal et al. (2012)
			TGF-β.		
		PRP+rBM-MSC	Promotes tendon repair and increase its structural strength.	Rat/Transverse cut	Yuksel et al. (2016).
		PRP+TSCs	Improves tendon healing**.**	Rat/3-mm long segment was removed	Chen et al. (2012)
		PRP +MSCs	Increases the inflammatory cell density and the mean maximum breaking force.	Rat/Cut	Uyar et al. (2022)
GFs/scaffolds	TGF-β	TGF-β1+BMSC	Improves tendon healing and tendon regeneration.	Rat/A defect of 5 mm in length and 1 mm in width	Zhang et al. (2018a).
		+collagen sponge			
		TGF-β3+PLGA-b-PEG NPs	Sustained-release TGF-β3 accelerates AT healing and remodeling.	Rat/A 3–4 mm gap	Cetik et al. (2022).
		TGF-β +Fibrin glue	Promotes fibrocartilage formation, the tensile strength.	Rabbit/Transect	Kim et al. (2007)
		TGF-β1+BMP12+bFGF	Increases mechanical strength and tendon remodeling.	Rat/Transect	Majewski et al. (2018).
		+collagen sponge			
	BMP				
		BMP-12+mineral-coated sutures/collagen sponge	Improves collagen organization, reduce adhesions, and decrease total cell numbers.	Rat/Transect	Chamberlain et al. (2015)
		BMP-2+fibrin glue	Accelerates bone-tendon healing, and improves histological/biomechanical properties.	Rabbit/Transect	Kim et al. (2007)
		BMP-12+collagen	Increases biomechanical properties, spindle-shaped fibroblasts.	Rat/A defect 6 mm in length	Xiao et al. (2021)
		BMP-2+PRP+fibrin	Accelerates bone-tendon junction repair, and increase the junction holding strength.	Rabbit/Transect	Kim et al. (2011).
		BMP-2+Osteoprotegerin	Increases Col I and Col II, promote fibrocartilage attachment.	Rabbit/ACL reconstruction	Wei et al. (2023)
		+collagen sponge			
	IGF	IGFBP4+PLLA electrospun membrane	Sustained release of IGFBP-4 promotes tendon healing in functional performance, ultrastructure, and biomechanical properties.	Rat/Transect	Wang et al. (2023)
	FGF	bFGF+BMP-12+TGFβ1	Increases mechanical strength and tendon remodeling.	Rat/Transect	Majewski et al. (2018).
		+collagen sponge			
		bFGF+VEGFA+nanoparticle-coated sutures	Enhances tendon healing, improves tendon gliding function,	Rat/Transect	Zhou et al. (2021b)
			Inhibits adhesion.		
		bFGF+VEGF+nanostructured mineral coating	Increases vascularization, collagen fiber organization, and mechanical properties, improve functional healing.	Rabbit/Transect	Yu et al. (2017)
		bFGF-fibrin gel +PLGA	Stimulates the proliferation and tenogenic differentiation of MSCs and synergistically enhance the injured tendon reconstruction.	Rat/A defect of 7 mm in length	Zhao et al. (2019)
		bFGF+ GPH nanofiber membranes	Provides a niche for inducing tendon tissue regeneration, and restoring the tendon tissue structure and function.	Rabbit/15-mm defect	Darshan et al. (2022)
		bFGF+ PCL micro/collagen	Enhances cellular proliferation and tenogenic marker expression, stimulates tissue regeneration with aligned collagen morphology.	Rabbit/Defect	Jayasree et al. (2019)
	PDGF	PDGF-BB+electrospun DegraPol^®^ tub	Accelerates tendon wound healing, causing a more uniform cell distribution with higher proteoglycan content and less fibrotic tissue; Increases the tensile strength, cell hyperproliferation, and α-smooth muscle actin expression at the wound site.	Rabbi/sliced	Meier Burgisser et al. (2020)
		PDGF-B+mesoporous silica nanoparticles	Accelerates AT healing.	Rat/NA	Suwalski et al. (2010)
		PDGF+microneedle patch	Enhances tendon healing quality, angiogenesis, stiffness, maximum load, and stress.	Rat/Transect	Liu et al. (2022)
		PDGF-BB+ DegraPol tube	Decrease adhesion formation.	Rabbit/sliced perpendicularly	Burgisser et al. (2021)
	VEGF	VEGF+bFGF+nanostructured mineral coating	Improves vascularization, collagen fiber organization, mechanical properties, and functional healing.	Rabbit/Transect	Yu et al. (2017)
	PRP	Bone-tendon quadriceps tendon graft+PRP	Enhances healing capability.	Patient/Chronic tendinosis	Kirschner et al. (2021)
		PRP+decellularized bovine tendon sheets	Remodel and integrate into the AT and improves the healing process	Rabbit/Transect	((Zhang et al., 2018b)
		PRP+ASCs+ hydrogel	Increases total photon flux, Mean ultimate failure load, ECM, and cellularity.	Rat/0.5- mm in width and 5-mm in length defect	Chiou et al. (2015)
Summary	Up to date, the tissue engineering technology combined with GFs, stem cells and scaffolds has been widely applied *in vitro* and *in vivo* studies of AT repair, and showed a superior therapeutic effect, which may be a promising treatment method for AT repair. But further studies need to be carried out to determine the optimal scheme.				

## 6 Clinical applications and limitations of GFs

GFs play a critical role in AT injury repair. GFs accelerate wound site restoration and improve functional recovery. However, they have some limitations, such as short effective half-life, instability, and promotion of scar and adhesion formation. PRP is the only GF to enter clinical trials for AT repair because of its apparent advantages, such as self-sufficiency, convenient extraction, and high safety. Based on the results of many basic research and animal experiments, some clinicians treated AT injury patients with local injections of autologous PRP. Nonetheless, the conclusions drawn from numerous clinical studies on the efficacy and safety of PRP remain inconsistent (Table 4). The three different isoforms of TGF (TGF-β1, -β2, and -β3) upregulate the production of Col I and Col III, playing an essential role in tendon healing. Contrarily, TGF-β1 appears to be responsible for scar and adhesion formation. A clinical trial of TGF-β for indications outside of the tendon had to be halted due to excessive scar formation; therefore, TGF-β was not tested in AT healing in the clinic. PDGF-AA drives both tenogenesis and fibrosis. However, further studies regarding using PDGF-AA to accelerate AT injury recovery while reducing scars are warranted. VEGF plays a vital role in angiogenesis during AT healing, and vascular ingrowth is necessary for tendon healing. However, hypervascularization may be associated with degeneration. Therefore, the role of GFs in AT repair is a double-edged sword, and the question of how to better apply it remains unsolved. As previously indicated, there is still a long way from basic research to clinical application.

**TABLE 4:** The clinical application of PRP for AT healing.

Growth factors	Application	Outcome	References
PRP			
	PRP	Not superior to placebo treatment in chronic AT diseases.	Liu et al. (2019); Boesen et al. (2020); Keene et al. (2022)
	PRP	Improves short and midterm functional outcomes in acute AT rupture.	Zou et al. (2016)
	PRP	Enhanced the maturity of the healing tendon tissues, promotes better ColI deposition, decreases cellularity, less vascularity, and higher glycosaminoglycan content.	Alsousou et al. (2015)
	PRP	Modest improvement in functional outcome measures, MRI appearance of diseased AT remained largely unchanged.	Owens et al. (2011)
	PRP	Not result in greater improvement in pain and activity.	de Vos et al. (2010)
	PRP	Injecting PRP for the treatment of chronic midportion Achilles tendinopathy does not contribute to an increased tendon structure or alter the degree of neovascularisation, compared with a placebo.	de Vos et al. (2011)
	PRP	PRP is not useful for the treatment of AT ruptures.	Schepull et al. (2011)
	PRP+endoscopyassisted percutaneous	did not yield superior functional and clinical outcomes.	Hung et al. (2022)
	PRP+Endoscopic debridement	The addition of PRP did not improve outcomes compared to debridement alone.	Thermann et al. (2020).
	FD-PFC+surgery	The patient could return to play at the pre-injury level at 3 months after surgery of rupture.	Morimoto et al. (2021)

## 7 Conclusion

Repair and healing after AT injury are low due to the tendon’s low cellularity, vascularity, and metabolic activity. In general, injured tendons rarely regain the structural integrity and mechanical strength of healthy tendons and are, therefore, prone to reinjury. GFs play a significant role in natural tendon regeneration as they participate in cell recruitment and stimulation of ECM synthesis. In recent years, GFs have been a popular treatment option for tendon injuries, which provided new perspectives on the healing of tendon injuries. The combination of multiple GFs with stem cells/scaffolds/other GFs also showed promising efficacy compared with individual GFs.

The GFs discussed in this review have important and varied roles in AT healing, affecting their functions and molecular changes. TGF-β2, IGF-I, and bFGF are involved in multiple signaling pathways and promote the synthesis of ECM components, cell proliferation, migration, and directed differentiation of MSCs. VEGF regulates angiogenesis within the wound site. PDGF shows different effectiveness in promoting tendon healing in varying stages of the injury. TGF-β2 and BMP-2 sometimes hinder AT injury recovery and even cause more severe complications. The effectiveness of PRP in AT repair remains controversial. There are also complex interactions between different cytokines, showing synergistic or antagonistic effects. Although clinical studies have confirmed the definite efficacy of some GFs, insignificant therapeutic effects have also been reported.

Several challenges must be overcome before GF delivery can translate into clinical practice, and further research is warranted (Prabhath et al., 2018). For instance, it is important to (1) further define the roles of each GF in AT healing and selecting the optimal GF(s) or a combination of GFs, (2) identify and clarify the synergistic and antagonistic influences they have on each other when combined, (3) confirm the most efficient stage and duration of delivery of each GF, (4) determine the effective dose of the selected GF and its retention time at the injured site, and (5) determine the most appropriate strategy for GF administration, including using gene therapy, recombinant GFs, or biomaterial scaffolds. If biomaterial scaffolds are used for GF sustained release, the most appropriate material and scaffold form must be selected.

Adhesion and fibrosis, which are critical problems affecting the degree of healing, are essential difficulties to overcome in repairing AT injury. GFs can simultaneously promote healing and cause fibrosis. The direction for further research should be how to effectively use GFs or combine GFs/stem cells/tissue engineering scaffolds in AT repair.
