# Supplementary material for: Growth factors in the treatment of Achilles tendon injury
Source: Front Bioeng Biotechnol. 2023 Sep 14;11:1250533. doi: 10.3389/fbioe.2023.1250533 (PMC10539943; doi:10.3389/fbioe.2023.1250533)
Supplement: Supplementary file 1 [file Table1.docx]

**Table s1 The role of growth factors during AT healing phases**

| GFs | Reference | Animals | Model  Establishment | Timepoint after injury | Outcomes | Conclusion |
| --- | --- | --- | --- | --- | --- | --- |
| TGF-β | (Majewski et al. 2012) | SD rats | Transection | 1/2/4/8w | 2w: AT increases in thickness and has a  nearly normal histological  appearance. Col Ⅰ was the main  fibers in the defects.  8w: The thickness of AT decreases and the mechanical properties increases significantly. | TGF-β reduces inflammation, inhibits tissue adhesion, promotes mechanical strength, regulates collagen synthesis, cross-link formation, and matrix remodeling.  Such as TGF-β2. |
|  | (Hou et al. 2009) | New Zealand White rabbits | Defect | 1/2/4/8w | 2w: Stimulates Col Ⅲ synthesis.  8w: Increases Col Ⅰ content. |  |
|  | (Kashiwagi et al. 2004) | Wistar rats | Transection | 1/2/4w | 1w: Col Ⅰ and Col Ⅲ mRNA increases  　　　dose-dependently.  4w: FL and stiffness of the healing  tendon increases. |  |
|  | (Huang et al. 2020) | SD rats | Transection | 2w | 2w: More Col III and less Col I in the  　　　AT.  　　　Increases inflammatory response, and  　　　decreases adhesion. |  |
| BMP | (Majewski et al. 2008) | SD rats | Transection | 1/2/4/8/12w | 1w: The maximum failure load (MFL)  　　　 increases.  1\2\4w: The AT stiffness increases.  8w: The AT thickness decreases and no  　　　 inflammation. | BMP accelerates AT healing, influences early tissue regeneration,  and improves biomechanical properties.  such as BMP-12  and BMP-14. |
|  | (Bolt et al. 2007) | SD rats | Transection | 1/2/3w | 2w： Most of the AT has robust healing with little or no gaping at the site of repair.  1\2\3w: AT exhibits the highest tensile  strength after surgery. |  |
| PDGF | (Shah et al. 2013) | SD rats | Collagenase induces  tendinopathy | 7/21d | 7d: The cells increase dose-dependently; tendon thickness increases at the calcaneal insertion; The MLF increases in 10.2 mg rhPDGF-BB group;  21d: The MFL increases significantly. | PDGF-BB increases cell proliferation and improves biomechanical properties in a dose-dependent manner. |
|  | (Solchaga et al. 2014) | SD rats | Collagenase induces  tendinopathy | 7/21d | 7d: The MFL significantly increases in  3 mg rhPDGF-BB group;  21d: MFL and stiffness increases in the  10 mg rhPDGF-BB group. |  |
| VEGF | (Zhang et al. 2003) | SD rats | Transection | 1/2/4w | Improves tensile strength early in AT healing. | Exogenous VEGF significantly improves tensile strength in the early phase of AT healing. |
| bFGF | (Najafbeygi et al. 2017) | New Zealand white rabbits | Transection | 42d | Improves the healing process, enhances  collagen orientation, and increases  bio-mechanical resistance. | bFGF promotes collagen synthesis and deposition, improves collagen fiber arrangement and nucleus morphology, and enhances the mechanical properties of the AT. |
|  | (Herbst et al. 2019) | Lewis rats | Defects | 1/2/3/14d | Increases the stiffness of the AT. |  |
| IGF | (Usami et al. 1988) | SD rats | Transection | 2d/15d | Reduces maximum functional deficit, and accelerates recovery of AT injury. | IGF reduces adhesion and improves the mechanical properties of the AT, including MLF, stiffness, and ultimate stress. IGF reduces the maximum functional deficit and accelerates recovery after AT injury by anti-inflammatory. |
|  | (Tang et al. 2015) | SD rats | Transection | 2/8w | 2w: The granulation tissues and inflammatory-cell infiltration are lighter.  8w: The scars of AT are less evident.  The adhesion scores are the lowest. 2/8w: MLF, stiffness, and ultimate stress of  healing AT is highest at 2/8w. |  |
| PRP | (Takamura et al. 2017) | Japanese albino rabbits | Transection | 1/2/3/4/6w | 2w: Increase fibroblasts and new blood vessels.  6w：Increases Col I content. | PRP therapy shortens the inflammatory phase in AT injuries, accelerates the healing process, and promotes scar tissue with better histological quality. |
|  | (Lyras et al. 2009) | New Zealand White rabbits | Defects | 1/2/3/4w | 1-2w (Inflammatory and proliferation phase): New blood vessels number increases, better-organized collagen fibers.  4w: Newly formed vessels reduce, and the healing process shortened. |  |
|  | (Aguilar-Garcia et al. 2018) | sheep | Transection | 2w | 2w: Decrease the inflammatory reaction. |  |
|  | (Fernandez-Sarmiento et al. 2013) | sheep | Transection | 4/8w | 4w: Densely and regularly arranged collagen fibers, lower density fibroblasts.  8w: Mature organization of collagen bundles, lower fibroblast, and vascular density. |  |
|  |  |  |  |  |  |  |

Note: This table summarizes articles on the treatment of AT injury by GF alone, and describes its effects at different stages, most of these studies are not described in the manuscript.

**Reference**

Aguilar-Garcia D, Fernandez-Sarmiento JA, Granados MD, Morgaz J, Navarrete R, Carrillo JM, Vilar JM, Cugat R, Dominguez JM. 2018. Effect of plasma rich in growth factors on the early phase of healing of surgically severed Achilles tendon in sheep: histological study. *J Appl Anim Res* **46**: 471-478.

Bolt P, Clerk AN, Luu HH, Kang Q, Kummer JL, Deng ZL, Olson K, Primus F, Montag AG, He TC et al. 2007. BMP-14 gene therapy increases tendon tensile strength in a rat model of Achilles tendon injury. *J Bone Joint Surg Am* **89**: 1315-1320.

Fernandez-Sarmiento JA, Dominguez JM, Granados MM, Morgaz J, Navarrete R, Carrillo JM, Gomez-Villamandos RJ, Munoz-Rascon P, Martin de Las Mulas J, Millan Y et al. 2013. Histological study of the influence of plasma rich in growth factors (PRGF) on the healing of divided Achilles tendons in sheep. *J Bone Joint Surg Am* **95**: 246-255.

Herbst E, Imhoff FB, Foehr P, Milz S, Plank C, Rudolph C, Hasenpusch G, Geiger JP, Aneja MK, Groth K et al. 2019. Chemically Modified Messenger RNA: Modified RNA Application for Treatment of Achilles Tendon Defects. *Tissue Eng Part A* **25**: 113-120.

Hou Y, Mao Z, Wei X, Lin L, Chen L, Wang H, Fu X, Zhang J, Yu C. 2009. The roles of TGF-beta1 gene transfer on collagen formation during Achilles tendon healing. *Biochem Biophys Res Commun* **383**: 235-239.

Huang S, Xiang X, Qiu L, Wang L, Zhu B, Guo R, Tang X. 2020. Transfection of TGF-beta shRNA by Using Ultrasound-targeted Microbubble Destruction to Inhibit the Early Adhesion Repair of Rats Wounded Achilles Tendon In vitro and In vivo. *Curr Gene Ther* **20**: 71-81.

Kashiwagi K, Mochizuki Y, Yasunaga Y, Ishida O, Deie M, Ochi M. 2004. Effects of transforming growth factor-beta 1 on the early stages of healing of the Achilles tendon in a rat model. *Scand J Plast Reconstr Surg Hand Surg* **38**: 193-197.

Lyras DN, Kazakos K, Verettas D, Polychronidis A, Tryfonidis M, Botaitis S, Agrogiannis G, Simopoulos C, Kokka A, Patsouris E. 2009. The influence of platelet-rich plasma on angiogenesis during the early phase of tendon healing. *Foot Ankle Int* **30**: 1101-1106.

Majewski M, Betz O, Ochsner PE, Liu F, Porter RM, Evans CH. 2008. Ex vivo adenoviral transfer of bone morphogenetic protein 12 (BMP-12) cDNA improves Achilles tendon healing in a rat model. *Gene Ther* **15**: 1139-1146.

Majewski M, Porter RM, Betz OB, Betz VM, Clahsen H, Fluckiger R, Evans CH. 2012. Improvement of tendon repair using muscle grafts transduced with TGF-beta1 cDNA. *Eur Cell Mater* **23**: 94-101; discussion 101-102.

Najafbeygi A, Fatemi MJ, Lebaschi AH, Mousavi SJ, Husseini SA, Niazi M. 2017. Effect of Basic Fibroblast Growth Factor on Achilles Tendon Healing in Rabbit. *World J Plast Surg* **6**: 26-32.

Shah V, Bendele A, Dines JS, Kestler HK, Hollinger JO, Chahine NO, Hee CK. 2013. Dose-response effect of an intra-tendon application of recombinant human platelet-derived growth factor-BB (rhPDGF-BB) in a rat Achilles tendinopathy model. *J Orthop Res* **31**: 413-420.

Solchaga LA, Bendele A, Shah V, Snel LB, Kestler HK, Dines JS, Hee CK. 2014. Comparison of the effect of intra-tendon applications of recombinant human platelet-derived growth factor-BB, platelet-rich plasma, steroids in a rat achilles tendon collagenase model. *J Orthop Res* **32**: 145-150.

Takamura M, Yasuda T, Nakano A, Shima H, Neo M. 2017. The effect of platelet-rich plasma on Achilles tendon healing in a rabbit model. *Acta Orthop Traumatol Turc* **51**: 65-72.

Tang Y, Leng Q, Xiang X, Zhang L, Yang Y, Qiu L. 2015. Use of ultrasound-targeted microbubble destruction to transfect IGF-1 cDNA to enhance the regeneration of rat wounded Achilles tendon in vivo. *Gene Ther* **22**: 610-618.

Usami M, Kotake T, Matsuda M, Okajima E, Osafune M, Akaza H, Isurugi K, Niijima T, Aso Y, Araki T et al. 1988. [Endocrine therapy of prostatic carcinoma with slow release (depot) formulation of the LH-RH analog ICI 118630 (Zoladex)]. *Hinyokika Kiyo* **34**: 369-382.

Zhang F, Liu H, Stile F, Lei MP, Pang Y, Oswald TM, Beck J, Dorsett-Martin W, Lineaweaver WC. 2003. Effect of vascular endothelial growth factor on rat Achilles tendon healing. *Plast Reconstr Surg* **112**: 1613-1619.
